# The T2T Genome of the Domesticated Silkworm *Bombyx mori*

**DOI:** 10.3390/ijms252212341

**Published:** 2024-11-17

**Authors:** Wan-Shun Li, Ying-Dan Xiao, Jian-Qiu Liu, Sheng-Long Li, Yue Chen, Ya-Jing Xu, Xue Yang, Yan-Jue Wang, Zhi-Qing Li, Qing-You Xia, Kazuei Mita

**Affiliations:** 1 Integrative Science Center of Germplasm Creation in Western China (Chongqing) Science City, Biological Science Research Center, Southwest University, Chongqing 400715, China; liwanshun_leo@163.com (W.-S.L.); xyd2069472847@163.com (Y.-D.X.); jianqiu256@outlook.com (J.-Q.L.); xiaqy@swu.edu.cn (Q.-Y.X.); 2 Department of Bioinformatics, The Basic Medical School of Chongqing Medical University, Chongqing 400016, China; lishenglong@cqmu.edu.cn

**Keywords:** Silkworm (*Bombyx mori*), T2T genome, lepidopteran insect, Gene prediction

## Abstract

Genome sequences contain the fundamental genetic information that largely determines the biology of a species. Over the past 20 years, advancements in high-throughput sequencing technologies and bioinformatics tools have matured, facilitating genome assembly and ushering in the telomere-to-telomere (T2T) era. *Bombyx mori* is renowned as a silk-producing insect and serves as an important model organism extensively studied across various fields of biology. In this study, we present the first assembled T2T genome by integrating HiFi, ultra-long ONT, NGS, and Hi-C data. This assembly comprises 450,267,439 base pairs from 28 chromosomes and includes annotations for a total of 18,253 protein-coding genes. A completeness evaluation revealed that 99.1% of conserved single-copy genes were included, as determined by a BUSCO analysis. Furthermore, the consensus quality (QV) assessed through Merqury was recorded at 59.88. The proportion of repeat sequence achieved 60.77%, marking it as the highest reported value for *B. mori* to date. In comparison to previously published genomes, our assembly offers a more complete and higher quality representation, particularly concerning highly homologous tandem regions such as telomeres, rDNA clusters, and Gr family regions. Furthermore, our extensive experience in genome assembly, including sample preparation experience and assembly strategies to reduce complexity, will provide valuable references for other species aiming to achieve their own T2T genome assemblies.

## 1. Introductions 

In the past two decades, genomics has entered a period of significant advancement, driven by the development of high-throughput sequencing technologies. The availability of omics data has greatly facilitated progress in biological research. A species reference genome as basal buildings for genomics is essential for such studies. While an increasing number of species reference genomes have been released, certain complex genomic regions remain unexplored [1]. As of 2020, the first telomere-to-telomere (T2T) X chromosome of humans was reported [2], followed by the T2T assembly of human chromosome 8 [3], and subsequently, the publication of the complete human genome [4,5]. Additionally, other T2T genomes, including Arabidopsis [6,7], rice [8,9], banana [10], and maize [11], have also been published. These milestones signify the onset of an era characterized by T2T genomes [1,12,13]. Currently, T2T genomes represent a new standard for reference genomes; new T2T assemblies for various animals and plants are being released monthly. However, there remains a notable absence of published T2T genomes among insects despite numerous chromosome-level assemblies having been made available [14].

The silkworm *B. mori* is renowned for its silk production and has been domesticated in China over the past 5000 years while being reared globally today [15]. In 2004, Chinese and Japanese researchers sequenced its draft genome for the first time to unveil its genetic mysteries [16,17]. Four years later, an improved version was released that integrated two previously published drafts by International Silkworm Genome Consortium [18]; this reference genome has since been extensively utilized over the following decade in various studies addressing topics such as biogenetic diversity [15,19], mechanisms underlying sex determination [20,21,22,23], functions within important gene families [24], and Chromatin modification regulation [25]. It is not only applicable to silkworms but also to other insects and organisms [26,27,28,29,30]. However, significant missing regions have hindered research progress due to the difficulty in assembling complete tandem duplication gene domains. Consequently, researchers opted to utilize BAC sequences to complete a GR domain, including eight high-similarity GR tandem repeats [24]. By 2019, a new chromosome-level genome was released on Silkbase [p50T(silkbase)], integrating hybrid PacBio, NGS, and BAC data while still leaving 30 gaps [31]. In subsequent years, another chromosome-level genome was released on SilkDB 3.0 [p50(silkDB3)] [32]. Following this, four female silkworm genomes, p50T(zhang) [33], p50(Han) [34], p50ma [35], and Nichi01 strain [36] were released using longer-read platforms. Nevertheless, these genomes also contain unknown regions; specifically, the telomere and other tandem repeat regions are incomplete and lack solid evidence regarding their quality. In this study, we present the first T2T *B. mori* genome report. This assembly comprises 450,267,439 bp across 28 chromosomes (excluding W). We have successfully assembled the first insect T2T genome; our findings will provide valuable insights for future studies.

## 2. Result

### 2.1. Genome Sequence of B. mori

We sampled the pupa of Dazao strain and generated HiFi, ONT, NGS, and Hi-C data for assembling the Dazao genome (Table 1). We obtained a total of 39.5 Gb of high-quality HiFi data, corresponding to 85.8× genome coverage, after correcting and removing reads shorter than 5 Kb. Additionally, we obtained a total of 145.8 Gb of raw ultra-long ONT reads. Following quality checks and the elimination of short reads, we utilized 79.4 Gb (172.6×) for self-correction using CANU (v2.0) [37], resulting in 61.5 Gb (133.7×) of high-quality data for subsequent analyses. Furthermore, we generated 63 Gb (136.9×) of NGS data and 61.5 Gb (133.7×) Hi-C data using the MGISEQ2000 platform.

### 2.2. T2T Assembly of Z Chromosome and 27 Autosomes in B. mori

**Figure 1:**
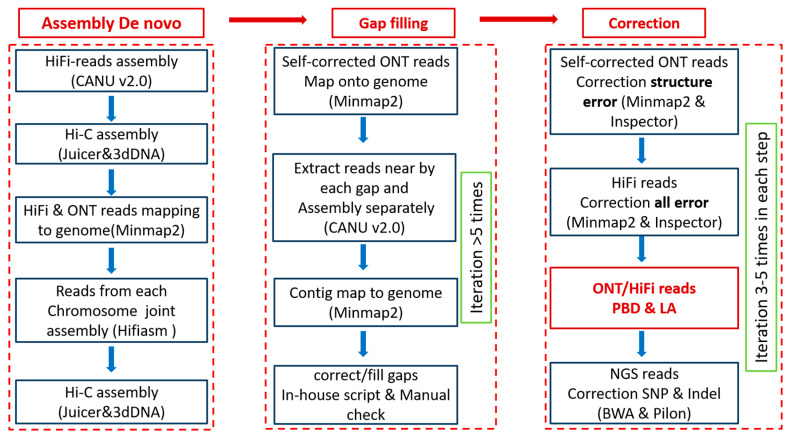
Overview of the genome assembly pipeline.

**Figure 2:**
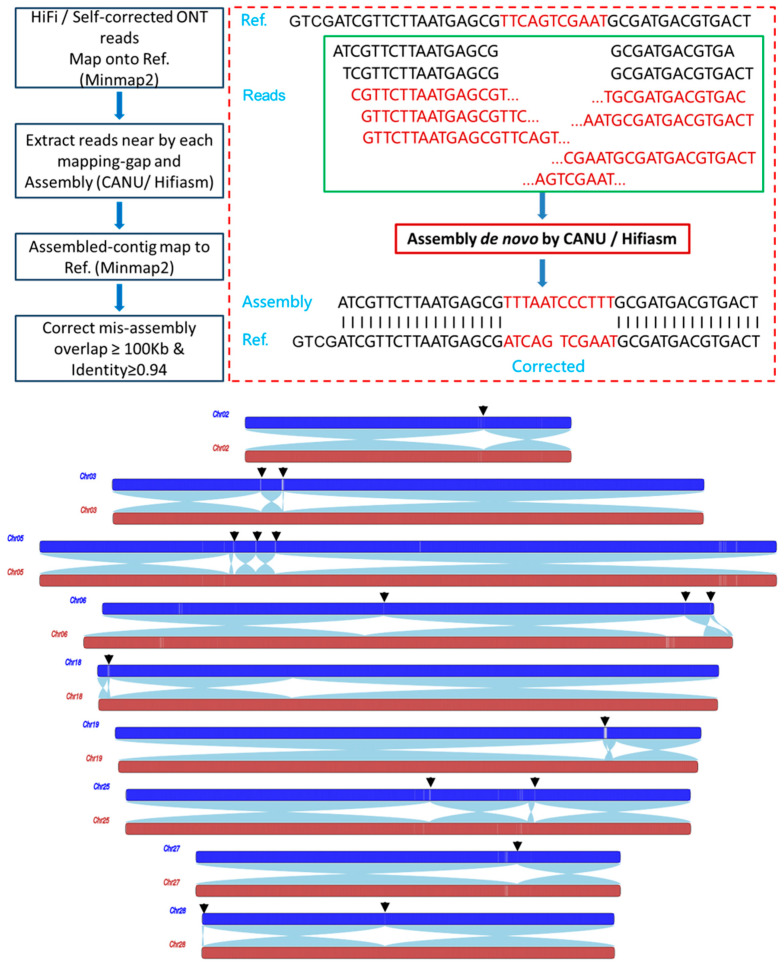
‘Perfectly mapped read-based assembly error detection (PBD)’ for quality improvement. Upper: an overview of the PDB method. The reads depicted in black represent end-to-end mapped reads, which will be utilized for quality assessment; conversely, the reads shown in red indicate partially mapped reads that may not align correctly. Both types of reads will be employed for assembly using CANU/hifiasm. Below: The reads coverage for “perfect-mapping reads” before and after correction through local assembly (LA). The blue and brown bars represent data prior to and following correction, respectively. The black arrow indicates the corrected position, while the white sections within the bar denote areas with low coverage. Notably, Chromosome 06 contains a ~900 kb missing region that has been identified and rectified. The sky-blue homolog block signifies positive collinearity, facilitating clear identification of specific breakpoints.

(1) Contig Assembly De Novo. The overview of our pipeline is summarized in Figure 1. Firstly, all HiFi reads were assembled de novo using CANU [37], yielding a primary contig assembly measuring 509.8 Mb in size. Next, a total of 107 contigs were classified into 28 chromosomes based on Hi-C data analysis. Eight contigs were fragmented due to insights from the Hi-C interaction map; notably, one contig was divided into three fragments originating from different locations. To simplify the dataset complexity and enhance assembly continuity, we mapped and extracted HiFi and ONT reads corresponding to each chromosome were then independently assembled by hifiasm [38]. Subsequently, the contigs were reallocated within their respective chromosomes utilizing additional information from the Hi-C data (Figure 1). This step successfully closed most gaps, but eighteen gaps still remained.

(2) Gap Filling by Local Assembly (LA) Using ONT Reads. All ONT reads were aligned with the initial assembly. The ONT reads located on a 100 kb region from each gap were extracted for independent assembly. Finally, the new assemblies were realigned back onto their respective chromosomes to fill existing gaps (Figure 1). This process was iterated several times until every gap was filled up. Some instances required manual judgment regarding gap filling or extension. Finally, all eighteen gaps and nearly half of telomeric regions were filled up. We successfully acquired all 56 telomere regions located at the ends of each chromosome, with an average length exceeding 10 kb.

(3) Correction through ONT or HiFi Read-to-Contig Alignment. Given that ONT reads are longer but have lower quality compared to HiFi reads, and NGS reads exhibit the highest quality among the three types, we initially employed ONT reads to correct structural errors using Inspector (V1.2) for three iterations. Subsequently, HiFi reads were utilized to rectify all types of errors with Inspector (V1.2) over another three iterations (Figure 1).

(4) Evaluation and Correction by PBD and LA. The read data contain native genomic information that can be utilized to assess assembly quality. However, current aligners only provide matches ranging from partial to full-length alignment. Generally, assembly errors predominantly occur in repetitive regions; thus, reads exhibiting partial alignments, rather than end-to-end matches, often arise when mapping onto homologous regions due to inaccuracies in the reference genome (Figure 2). Consequently, the evaluation of assembly quality based on the coverage of “full-length alignment” reads (perfectly mapped reads) is reasonable and effective. In this study, we introduced a method termed ‘perfectly mapped read-based assembly error detection (PBD)’ for estimating assembly quality. If the coverage of “full-length alignment” reads falls within a range of 0.5–2.5× the average depth across an entire chromosome, then the assembly is deemed correct; otherwise, it is classified as an incorrect linkage requiring correction.

Despite corrections made through the read-to-contig alignment method, our application of the PBD method revealed 28 regions with low read coverage indicative of assembly errors (Figure 2), which proved challenging to improve using read-to-contig alignment strategies alone. These erroneous regions were subsequently corrected individually by local assembly (LA). Notably, we detected a missing region spanning ~900 kb on Chr6; this may have resulted from incorrect connections within high-repeat areas (Figure 2). After correction, the read coverage returned to normal levels, and a corresponding region was identified by comparing it to the released *B. mori* genome.

On the other hand, regions with high read coverage indicate that some repeats within this domain may still be collapsed into fewer copies. This case is always induced by nearly identical region and bigger repeat units. Longer reads and a larger overlap parameter value of CANU (-minOverlapLength) can be employed over the high similarity and re-build the collapsed units.

Re-assembly of the rDNA Region. In the initial assembly version, the read depth of the rDNA region (Chr11) was three times higher than the average depth across the entire chromosome. Extracted 30× the longest ONT reads mapped to this region and performed assemblies using CANU [37] with parameters set at ‘-minOverlapLength=110,000′, which resulted in the reassembly of additional rDNA fragments. These seven fragments were ultimately located on Chr11 (4.375–6.499 Mb), encompassing approximately 2.1 Mb within a continuous region. This domain encompasses copies of ribosome elements, such as the 18S, 28S, and other rDNA elements (NCBI accession no M27187.1, XR_005246515.1, DQ347470.1 and M16558.1). The complete sequence of a ribosomal RNA gene (DQ347470.1) is exclusively reconstructed in DazaoT2T among publicly available genomes.

GRs cluster re-constructed. In 2017, Guo et al. [24] conducted a comprehensive survey of the Gustatory receptor (Gr) gene family across the entire genome. Among their findings, a significant cluster located on Chr07 was identified, which contains eight almost 100% identical copies (designated as Gr30-1 to Gr30-8) based on BAC sequencing. In the present study, we reassembled this region utilizing localized ONT reads processed through CANU with parameters set at ‘-minOverlapLength=37,000’, resulting in the identification of eight distinct copies of Gr30.

(5) Polish through NGS reads. Although HiFi and ONT reads exhibit high quality, their error rates remain higher than those associated with NGS [1]. Consequently, after employing PBD and LA methods for correction, minor errors still persisted; these were subsequently rectified using NGS reads through Pilon with three iterations of correction. The final T2T Dazao Z- and auto-some sequences were obtained, totaling 450,267,439 bp (accession numbers CNA0143014).

### 2.3. Evaluation of T2T Assembly

**Figure 3:**
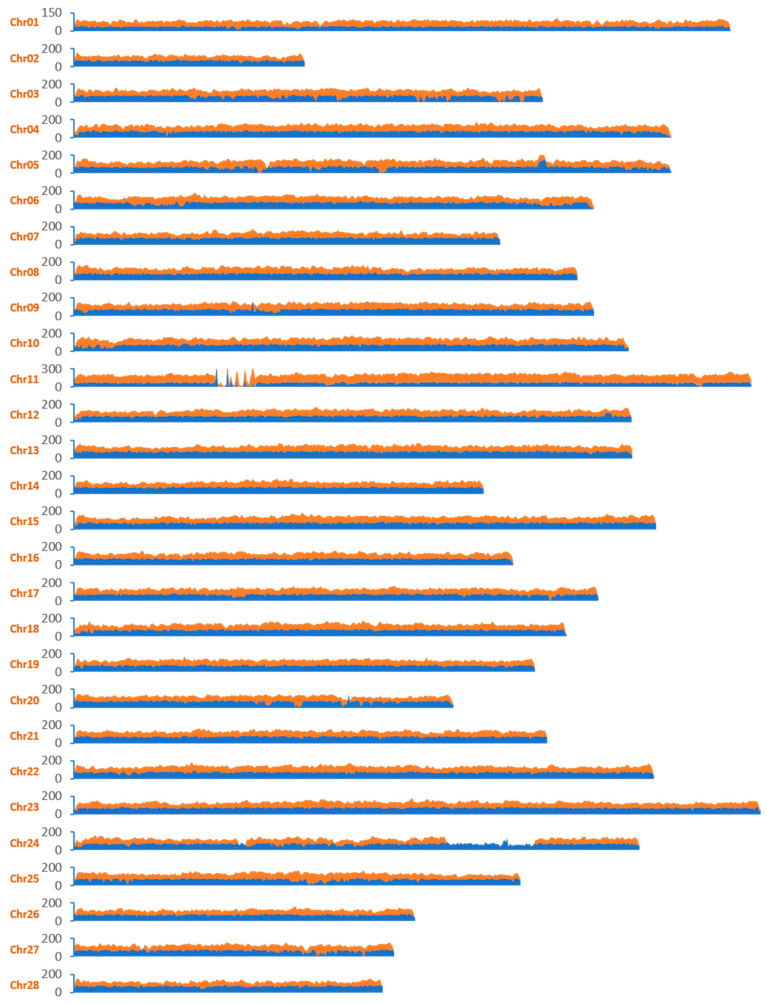
Whole-genome coverage of HiFi (blue) and ONT (orange) reads across the final assembly. Ultra-long ONT reads longer than 50 kb and HiFii reads longer than 5 kb were used for coverage analysis.

**Figure 4:**
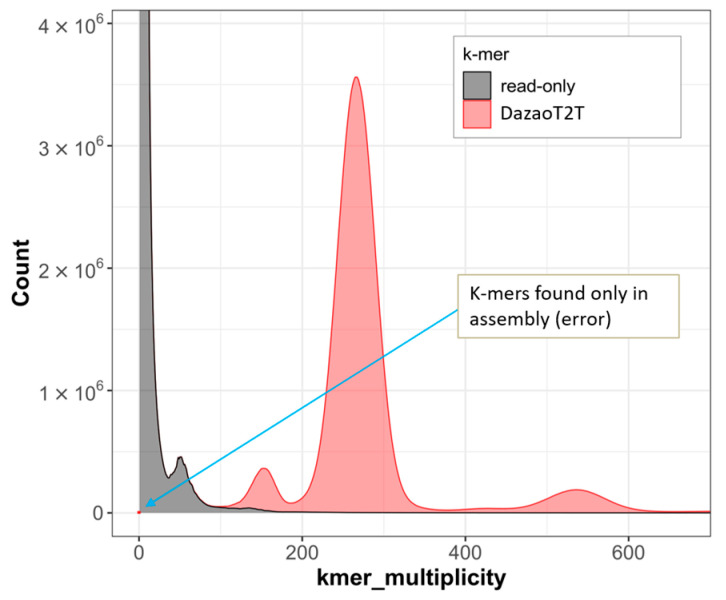
The quality of the DazaoT2T assembly assessment by Merqury. The spectrum plots of k-mers in assembly (red) and those that are read-only (gray) for the evaluation of assembly error and completeness. The X-axis represents the number of times a k-mer occurs in the read set.

**Figure 5:**
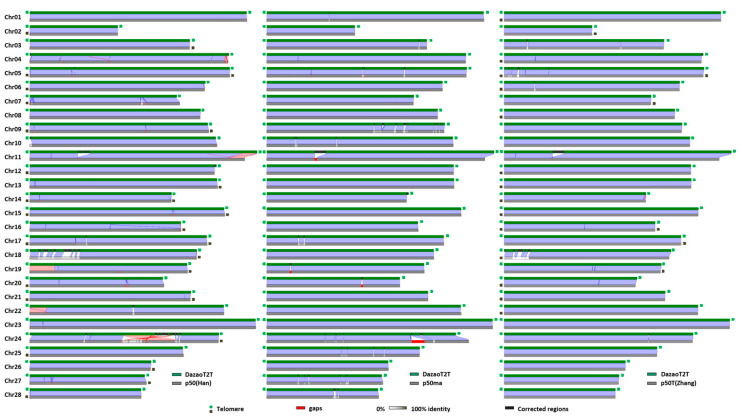
Improvements in DazaoT2T compared to the other three silkworm chromosome-level genome assemblies. Collinearity between the silkworm p50(Han) (**left**), p50Tma (**middle**), and p50T(Zhang) (**right**) is illustrated. Synteny blocks are represented by light blue lines, while inversions are indicated by red lines. Telomere sequence repeats are marked with light green and gray markers near the ends of the chromosomes. Red blocks denote existing gaps within the genome, whereas corrected gap regions in the other three assemblies are depicted as black blocks on the genome bar of DazaoT2T. The density of gray in the bar indicates homology to DazaoT2T, ranging from 0% to 100% identity.

(1) We calculated cumulative coverage for full-length mapped HiFi and ONT reads across all chromosomes. The results indicated that each chromosome was continuously covered by either HiFi or ONT reads; most regions exhibited coverage ranging from 100× to 200× with an average coverage of approximately 130× for ONT reads and around 75× for HiFi reads (Figure 3). Notably, Chr01 showed about half the depth compared to autosomes. Certain peaks in read coverage observed along chromosomes were primarily influenced by repetitive sequences, a phenomenon similarly noted in human T2T genomes [4]. A few regions displayed exclusive coverage by either ONT or HiFi reads; this discrepancy may stem from individual variations or missing during sequence process, as the genomic DNA were derived from distinct individuals reared by different batches. For instance, one example spans from 11 to 14 Mb on Chr24: this region is uniformly covered by HiFi reads but lacks any representation from ONT data—this area has also been found in other assemblies of *B. mori*.

(2) Merqury [39], a k-mer-based assessment pipeline, was employed to evaluate its completeness and assembly errors using all k-mers derived from NGS, HiFi, and ONT data. The QV score reached up to 59.88, while the QV scores for Chr26 even approached infinity, indicating no assembly errors (Table S1 and Figure S1). The read-only Kmers originate from sequence errors and a few heterozygous kmers (Figure 4).

(3) The presence of conserved single-copy genes serves as an additional index for estimating genome completeness. A total of 99.1% of single-copy BUSCOs from odb10 were identified in the current genome using BUSCO (v5.7.1) [40]. That is comparable to other chromosome-level genomes (Table 2).

(4) Compared to other assemblies among several indicators, including the total length of chromosomes, number of gaps, and BUSCO score, hints that p50ma, p50(Han), and p50T(Zhang) should be the three most complete genomes released; for example, they are most proximate to DazaoT2T and each other in total length and very close to the BUSCO score of genome completeness. Therefore, the collinearity analysis comparing DazaoT2T with each of these three assemblies showed that errors varying in degree and at different locations exist in the present genome, which are highlighted in Figure 5. Briefly, a large rDNA region (Chr11) is missing in all three genomes. Except for the rDNA region, two other high-frequency error regions have been found in Chr18 and Chr24. Five and one inversions were detected in p50(Han) and p50T(Zhang), respectively. Although no gaps were reported in the assembly of p50T(Zhang), at least 16 low-quality assembly regions have been detected comparison to others. Each improvement in DazaoT2T compared to each other one could be supported by another chromosome-level genome assembly, which suggests that DazaoT2T is a reliable assembly in the variation regions. Moreover, our PBD method for correction is reasonable and highly efficient.

(5) The collinearity analysis was conducted on a large scale; additionally, we also assessed it on a smaller scale, focusing on high-similarity tandem repeats of telomeres and gene families in tandem.

**Figure 6:**
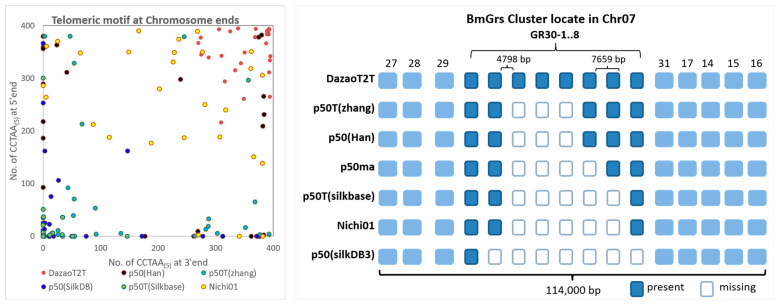
The completeness assessment comparison to other *B. mori* genome. (**Left**): The telomere length in each ends of chromosomes, the length represents numbers of CCTAA_(5)_ within each terminal 10 kb of chromosomes. p50ma: no CCTAA_(5)_ motif has been found in the ends of chromosomes. (**Right**): Detailed BmGrs cluster distribution in the Chr07. The blank rectangle represents the missed copies of Gr30 in the present genome. These eight Gr30 (size of 7659 bp) almost ‘cut/paste’ copies are almost 100% identical.

Telomeric sequences are frequently incomplete or entirely absent in existing assembled insect genomes. In this study, we successfully obtained all 56 complete telomere regions averaging over 10 kb in length. However, the published silkworm genome exhibits a lower completeness of telomere regions. The Nichi01 strain possesses the most complete telomeres, featuring 19 chromosomes, each with a pair of telomeres at both ends and a TTAGG(n) region that extends to at least 2.5k. Specifically, among the three highest completeness genomes, p50ma, p50(Han), and p50T(Zhang), with only zero, six, and two chromosomes, respectively, display complete telomeric sequences at both ends (see Table 2 and Figure 6, left).

Moreover, we successfully reconstructed all eight nearly 100% identical copies of the GRs cluster located on Chr07. The sequence assembled for this region exhibits over 95% identity with the BAC sequence (accession number LC056060 in DDBJ). In comparison to other published genomes, both p50 (Han) and p50T (Zhang) identified five Gr30s, making it the second most frequently reconstructed copy (Figure 6, right).

Overall, these data indicate that a high-quality T2T assembly of the *B. mori* genome has been achieved to date. This represents the first complete T2T insect genome, which includes fully resolved telomeres and demonstrates reliable quality comparable to other T2T genomes from humans and various plants known to us.

### 2.4. Annotation

Repeats. By integrating homology-based approaches with de novo predictions, repeat sequences comprise 60.77% of the *B. mori* genome. In terms of superfamily classification, LINE elements represent the largest proportion at 38.94%, followed by DNA repeats at 15.16%, SINEs at 13.09%, and LTRs at 12.04%. Chromosome-level analyses revealed that Chr1 (Z) exhibited the lowest proportion of repeats at 48.09%, whereas chr2 displayed the highest proportion at 75.59%. Notably, the GC content within chromosomes demonstrated a positive correlation with the proportion of repeat sequences (Figure S1).

Protein-coding genes. In this study, we integrated existing *B. mori* gene sets along with RNA-seq data to annotate proteins in T2T genomes using GeMoMa (v1.9) [41]. This software facilitates gene prediction by employing intron models derived from homologous gene sets. The candidate gene sets, ‘SilkbaseSet’ and ‘SilkDBSet’, were predicted based on references from silkworm gene sets available in Silkbase and SilkDB3.0 databases, respectively. Completeness was assessed using BUSCO (v3.0.2) [40], revealing that SilkbaseSet demonstrated higher integrity; thus, it served as our foundational gene set while adding high-quality genes exclusive to SilkDBSet into our final set. Genes were supported only by RNA-seq data (with a minimum coverage value no less than 2 in GeMoMa output). Ultimately, we identified a total of 18,253 protein-coding genes within the whole-genome analysis. The results of the BUSCO evaluation showed that the integrity of the gene set was reaching 95.8%, which was better than the currently known gene set of *B. mori* (Table 2).

## 3. Methods

### 3.1. Sample Preparation and Genome Sequencing 

The *B. mori* strain (Dazao) was reared and maintained in the SWU laboratory, China. The larvae were fed fresh mulberry leaves under standard conditions of a 12 h light and 12 h dark cycle at 25 °C. To minimize the phylogenetic background, single female–male pairs were mated, with each female’s eggs being reared in a separate container. Pupae were subsequently sampled for sequencing. Genomic DNA was extracted from a single female pupa, followed by library construction and sequencing on the PacBio platform using the HiFi sequencing model (HiFi, Sequel II) as well as on the NGS platform (MGISEQ-2000) at BGI-Tech (Wuhan, China). Additionally, genomic DNA was extracted from another individual female pupa for ultralong reads with Oxford Nanopore Technologies (ONT), utilizing PromethION at Nextomics Biosciences (Wuhan, China). Raw ONT reads with an average base quality below 7 or length shorter than 50 Kb were discarded; remaining reads underwent self-correction via CANU (v2.0) [37] with the following parameters: genomeSize = 900m -minOverlapLength = 21,000 -corOutCoverage = 200 “batOptions = -dg 3 -db 3 -dr 1 -ca 500 -cp 50” rawErrorRate = 0.4. The self-corrected ONT reads served as the basis for all analyses conducted in this study. A Hi-C library was constructed from approximately ten mixed female pupae using MboI restriction enzyme digestion; sequencing was performed on the MGISEQ platform, while low-quality reads were removed at BGI-Tech.

### 3.2. Contig De Novo Assembly Using HiFi and ONT Reads

The HiFi reads was assembled using CANU (v2.0) [37] with the following parameters: -minOverlapLength = 1000 -minReadLength = 5000 -corOutCoverage = 80 “batOptions = -dg 3 -db 3 -dr 1 -ca 500 -cp 50”.

Ultra-long self-corrected ONT reads was assembly through CANU (v2.0) [37], employing these parameters: -minOverlapLength = 19,000–minReadLength = 50000–corOutCoverage = 80 “batOptions = -dg 3 –db 3 -dr 1 -ca 500 -cp 50”. 

The joint assembly of HiFi and ONT reads were performed by Hifiasm (v0.19.9-r616) [38] using the following parameters: -k 59 -N 80 --ul-rate 0.02.

### 3.3. Chromosome Construction Using Hi-C Data 

To sort contigs for each chromosome, chromosome construction is performed utilizing Hi-C data. The process involves the following steps: (1) Each end of the Hi-C reads was mapped to the genome separately using BWA (v0.7.12) [42], followed by combining the pair-end realignment results. (2) The output was extracted and sorted according to experimental expectations using Juicer (v1.6) [43]. (3) An interaction map was constructed employing 3D-DNA (v180419) [44]. (4) Finally, a manual review was conducted to adjust fragments with incorrect positions and classify chromosomes via Juicebox (v1.11.08) [43].

### 3.4. Gap Closure Through Local Assembly (LA)

To fill gaps with HiFi, the HiFi reads were mapped onto the chromosome with Minimap2 (v2.28) [45] utilizing the following parameters: -k19 -w11 -g8000 --max-chain-skip 5 -r500 -m6000 -A1 -C9 -O6,26 -B4 -E2,1 -s200 -z200 -N5 --min-occ-floor = 500. Subsequently, reads were extracted for assembly that are located within a 50 kb region around each gap/end and have homologous regions ≥ 8 kb in length with an identity ≥ 80%. Reads extracted at each gap were assembled individually using CANU (v2.0). Contigs are extended or corrected when homologous matches between new assemblies and references met these criteria: overlap ≥ 80 kb and identity ≥ 95%.

Additionally, gaps were also filled up using ONT reads; these reads were mapped onto the reference genome with Minimap2 (v2.28) [45] under similar parameters as follows: -k19 -w11 -g20000 --max-chain-skip 6 –r500 –m16000 –A1 –C9 –O6,26 –B4 –E2,1 –s200 –z200 –N5 --min-occ-floor = 500. Reads for assembly were then extracted from a region of up to 200 kb near each gap/end where homologous lengths ≥ 18 kb and identities ≥ 80%. Each gap/end underwent separate assembly through CANU (v2.0). Corrections or extensions occurred when new assembly contigs matched references achieving overlaps of at least ≥100 kb and identities reaching at least ≥94%.

### 3.5. Perfectly Mapped Read-Based Assembly Error Detection (PBD) 

To assess the quality of the assembly, HiFi and ONT reads were aligned to the chromosome using Minimap2 (v2.28) [42], permitting only primary alignments and “full-length alignment” reads. The parameters for Minimap2 included “--secondary=no”, while all other settings remained consistent with those previously described. In this study, “full-length alignment” reads are defined as HiFi reads permitted to have non-matching regions of up to 200 bp at each end, while ONT reads are allowed no more than 1 kb of non-matching regions at both ends. If the coverage of these “full-length alignment” reads ranged from 0.5× to 2.5× the average depth across the entire chromosome, the assembly was deemed correct; otherwise, it was classified as a poor linkage and would be corrected through LA (as described in Method 4).

### 3.6. Correction of Assembling Errors Using Read-to-Contig Alignment

The ONT and HiFi reads were mapped to the entire genome using Minimap2 (v2.28) with the same parameters as PBD. Subsequently, ONT reads were processed for structural error correction using Inspector (V1.2) [46] with default parameters, while HiFi reads were utilized for all types of errors through Inspector (V1.2) with its default settings. 

NGS reads were aligned using BWA (v0.7.12) [42], followed by small error correction utilizing Pilon (v1.23) [47] with the following parameters: --diploid --duplicates --minmq 40 --mindepth 27 --minqual 0 --threads 3 --fix snps, indels, gaps. 

### 3.7. Genome Quality Evaluation

(1) Coverage of Reads evaluation. ONT/HiFi reads were aligned to the assembled sequence using Minimap2 (v2.28), and coverage for “full-length alignment” reads on the reference sequence was calculated based on criteria similar to those used in the PBD analysis. Read coverage was assessed within a sliding window of every 50 Kb (with a step size of 25 Kb) along each chromosome; however, regions within 20 Kb from both ends were excluded from consideration. 

(2) K-mer evaluation. The completeness of the final T2T *B. mori* assembly was estimated from mapped k-mers via Merqury (v1.1) [45], employing default parameters with NGS, HiFi, and ONT data serving as reference k-mers. 

(3) BUSCO evaluation. The completeness assessment of the assembly utilized BUSCO (Benchmarking Universal Single-Copy Orthologs) (v5.7.1) [40] against lepidoptera_odb10 as a reference dataset.

(4) Collinearity analysis. The released genome was aligned to the DazaoT2T sequence using Minimap2 (v2.28) with parameters as follows: --secondary=no -k19 -w11 -g20000 --max-chain-skip 1000 –r500 –m16000 –A1 –C9 –O6,26 –B4 –E2,1 –s200 –z200 –N5 --min-occ-floor=500. The homologous block at least 20kb was been retained for analysis. The figure was generated using the NGenomeSyn(v1.41) software program [48].

### 3.8. Repeat Annotation

In this study, the repeats in the T2T genome of silkworms were annotated using both ab initio prediction and homology alignment based on an existing repeat library. 

(1) De Novo Prediction of Repeats. I. Tandem Repeats Finder (TRF v4.10.0) [49] was employed to predict tandem repeats with the following parameters: 2 7 7 80 10 50 2000 -d -h. II. Ab initio prediction of LTR retrotransposons was conducted using LTR-Finder (v1.0.7) [50], applying the following parameters: -C -w 2 -s (Silkworm tRNA sequence library). Subsequently, any LTR sequences overlapping with tRNA were filtered out, and the remaining LTR sequences were extracted to create a reference database for de novo prediction. III. The transposon database was constructed utilizing RepeatModeler (v2.0.1) [51]. Initially, a seed database was established using the BuildDatabase tool with the following argument: -engine rmblast -name mydb. The repeat search was then performed using RepeatModeler [51] with the following parameters: -database mydb -pa 6. IV. The results from LTR-Finder and RepeatModeler were combined to form a final reference database, followed by another round of repeated sequence predictions and classifications using RepeatMasker (Version: open-4.0.7) [52]. This process utilized the following parameters: -nolow -no_is -norna -engine ncbi -parallel 1. 

(2) Homologous Prediction. Referring to known repeat sequence data across all species in RepBase [161], RepeatMasker (Version: open-4.0.7) [52] was used for DNA-level repeat sequence predictions employing the following parameters: -nolow -no_is -norna –engine ncbi –parallel 1 –lib; additionally, protein-level predictions were made with parameters set as follows: –engine ncbi –noLowSimple –pvalue 0.0001. These predictions were subsequently integrated to generate a comprehensive output of repetitive elements.

### 3.9. Coding Gene Annotation

In this study, we integrated homologous gene sets and RNA-seq data to annotate proteins in T2T genomes using GeMoMa (v1.9) [41], following the procedures described below.

(1) The RNA-seq data (RNAseq set) was aligned to the T2T genome using HISAT2 (v2.1.0) [53] with the following parameters: --dta -p 2 --sensitive --no-discordant --no-mixed -I 1 -X 1000; all other parameters were set to their default values. The RNA-seq data were obtained from Silkbase (accession numbers: DRR013821, DRR013822, DRR013823, DRR013824, DRR013825, DRR013826, DRR013827, DRR013828, DRR015667, and DRR015668).

(2) In the subsequent step, we combined the RNA-seq data with candidate gene sets (SilkbaseSet and SilkDBSet), which were predicted by GeMoMa [41] (v1.9). This prediction utilized silkworm gene sets from both Silkbase and SilkDB3.0 databases as references separately. GeMoMa employed the following parameters: CLI GeMoMaPipeline AnnotationFinalizer.r=NO p=false GAF.a=“iAA>=1” ERE.c=true tblastn=false; all other parameters remained at their default settings.

(3) Transposon factors were subsequently removed from our analysis. The nucleic acid sequences of both SilkbaseSet and SilkDBSet were extracted and translated into protein sequences. Functional annotation was performed using BLASTP software (NCBI-BLAST-2.9.0+) against NCBI’s non-redundant protein sequence database with an e-value threshold of ≤1 × 10^−6^. Any functional descriptions related to transposon factors were excluded from further consideration.

## 4. Discussion

Silkworm has garnered significant attention as a silk-producing bioreactor and a model organism in the Lepidoptera order. In this study, we present the first report of the T2T genome of *B. mori*, achieved through joint assembly that integrates HiFi, ultra-long ONT, NGS, and Hi-C data. We implemented a rigorous evaluation and correction pipeline (PBD) to enhance assembly quality by permitting only primary alignments and full-length alignment reads for assessing the accuracy of the assembly. Utilizing this pipeline, we identified 28 assembly errors across various regions; notably, one error involved a ~900 kb missing region that was rectified using long-read technology and validated through homologous alignment. Coverage evaluation indicated that each region is covered evenly by long reads, with coverage ranging from 0.5× to 2.0× relative to the average across whole chromosomes (Figure 3). The assembly quality value is 59.88, as assessed by Merqury [39]. In comparison to other released assemblies, especially to the three most completeness genome released p50ma, p50(Han), and p50T(Zhang), DazaoT2T obviously improved on a large scale, via collinearity analysis (Figure 5), and also on a small scale, via completeness of telomeres and Grs cluster (Figure 6). These data suggest that we have successfully obtained a high-quality T2T genome of *B. mori*, which, to our knowledge, represents the first T2T genome documented in insects thus far.

Nowadays, the T2T (telomere-to-telomere) genome represents a new standard for complete genomes. In insects, although more than 600 genomes have been published, there is still a lack of T2T genome publications. A T2T genome typically requires the integration of HiFi and ultra-long ONT data for assembly, followed by correction using NGS data; thus, an adequate amount of DNA is essential. The body size of an organism correlates with the quantity of DNA it contains. For most insects, the available DNA is often limited by the body size of individual specimens; therefore, multiple individuals must be combined to obtain sufficient DNA for sequencing. However, mixing DNA from several individuals can increase heterozygosity in genomic data, which poses challenges in assembling a high-quality genome. In this study, we utilized different individuals to extract DNA and generate genomic data through various platforms (Table 1). The results revealed significant genetic differences between genomes sequenced using HiFi and ONT data. For instance, in the ONT dataset, we observed a notable missing region on chromosome 24 (Figure 3), which was verified through homologous searches against published genomes—this discrepancy hindered our assembly process. Consequently, it is beneficial to extract as much DNA as possible from individual specimens and produce datasets across various platforms using identical DNA samples.

In eukaryotes, repetitive sequences constitute a major component distributed throughout the entire genome. Identical motifs of these repeats are often found at multiple loci with varying copy numbers. For instance, in *B. mori*, 60.77% of the genome sequence identified in this study consists of repeats, which is higher than that observed in most published insect genomes. This abundance of repeats complicates efforts to close all gaps within the genome. To mitigate this complexity and simplify assembly processes, we implemented a series of pipelines. We utilized Hi-C data to separate each chromosome and extracted reads for individual chromosome assemblies again; as a result, we successfully closed most gaps (81 out of 89 total gaps) based on the primary assembly. This approach represents an efficient strategy for addressing relatively low-complexity gaps early in the process. Furthermore, remaining gaps and errors were filled or corrected using long-read local assembly (LA), which employs only mapped reads adjacent to each gap for independent assembly—this method proves effective not only for filling gaps but also for extending telomeres. Additionally, longer reads significantly enhance gap-filling efficiency. These experiences will serve as valuable reference methods for other species and expedite the completion of T2T genomes in various insects.

## Figures and Tables

**Table 1:** The summary of sequence data from Dazao genome.

Dazao	PacBio HiFi (>5 Kb)	Self-Corrected Ultra-Long ONT (>50 Kb)	NGS	Hi-C
Pupa source	1 female	1 female	1 female	10 females
Data (Gb)	39.5	61.5	63.0	61.5
Coverage of genome (×)	85.8	133.7	136.9	133.7
N50	15,252	90,115	NA	NA

**Table 2:** Summary statistics of the genome assembly and predicted genes of the silkworm assemblies.

	DazaoT2T	p50ma	p50 (Han)	p50T (zhang)	Nichi01	p50T (silkbase)	p50 (silkDB3)
Num. of chromosomes	28	28	28	28	28	28	28
Total length of chromosome (kbp)	450,267	450,467	449,363	448,026	445,291	445,114	454,710
Num. of gaps	0	40 *	>25 *	16 *	4	30	729
Num. of chromosome with a pair of telomeres ^#^	28	0	6	2	19	0	1
Num. of chromosome with one of telomeres ^#^	0	0	14	14	9	2	8
The proportion of repeat sequence (%)	60.77	NA	57 [34]	NA	46.83 [36]	47.45 [31]	NA
BUSCO score of “single-copy” genes on chromosome (%)	99.1	99.1	99.1	99.1	99	99.1	98.9
BUSCO score of “duplicated” genes on chromosome (%)	0.3	0.3	0.8	0.4	0.8	0.9	1.8
BUSCO score of “fragmented” genes on chromosome (%)	0.4	0.4	0.5	0.4	0.5	0.5	0.5
Num. of predicted coding genes	18,253	NA	15,950	NA	18,397	16,880	16,069
BUSCO score of “single-copy” genes on predicted proteins (%)	95.4	NA	88.3	NA	94.7	91.6	77.7
BUSCO score of “duplicated” genes on predicted proteins (%)	0.8	NA	0.8	NA	1	1.2	1.9
BUSCO score of “fragmented” genes on predicted proteins (%)	1.8	NA	1.2	NA	1.2	3.5	6.3

* The number of gaps is from comparison to DazaoT2T (Figure 5); ^#^ the criterion for a complete telomere is defined as the presence of at least a 2.5 kb TTAGG(n) region, located in region of 10 kb from the chromosome end.

## Data Availability

All the raw sequencing data for this study have been deposited in the China National GeneBank (CNGB) Sequence Archive (CNSA) database under the accession code CNP0005783. The Dazao T2T assembly sequences (accession numbers CNA0143014).

## Supplementary Materials

The following supporting information can be downloaded at: https://www.mdpi.com/article/10.3390/ijms252212341/s1.
