# Supplementary material for: The T2T Genome of the Domesticated Silkworm Bombyx mori
Source: Int J Mol Sci. 2024 Nov 17;25(22):12341. doi: 10.3390/ijms252212341 (PMC11594454; doi:10.3390/ijms252212341)
Supplement: Supplementary file 1 [file ijms-25-12341-s001.zip › ijms-3298018-supplementary.pdf]

Table S1. The QV value assessment by Merqury.

Chr	length (bp)	K-mers in genome only	common K-mers in both	QV	error possiblity
Chr01	20702373	171	20702357	63.1347	4.86E-07
Chr02	8410284	9	8410268	72.0102	6.29E-08
Chr03	15280781	864	15280765	54.7807	3.33E-06
Chr04	19018323	128	19018307	64.0241	3.96E-07
Chr05	19017521	468	19017505	58.3935	1.45E-06
Chr06	16775540	273	16775524	60.1896	9.57E-07
Chr07	14042223	343	14042207	58.4259	1.44E-06
Chr08	16314353	2	16314337	81.4199	7.21E-09
Chr09	16821697	451	16821681	58.0214	1.58E-06
Chr10	17761834	233	17761818	61.1258	7.72E-07
Chr11	21704787	2725	21704787	51.3161	7.38E-06
Chr12	17837365	84	17837349	65.575	2.77E-07
Chr13	17878236	53	17878220	67.585	1.74E-07
Chr14	13548894	9	13548878	74.0811	3.91E-08
Chr15	18557905	6	18557889	77.2083	1.90E-08
Chr16	14425536	8	14425520	74.8649	3.26E-08
Chr17	16893552	122	16893536	63.7181	4.25E-07
Chr18	15944915	60	15944899	66.5492	2.21E-07
Chr19	15019242	2	15019226	81.0607	7.83E-09
Chr20	12704505	400	12704489	57.3234	1.85E-06
Chr21	15389305	21	15389289	70.9545	8.03E-08
Chr22	18521747	46	18521731	68.3537	1.46E-07
Chr23	21555315	29	21555299	71.016	7.91E-08
Chr24	18056436	184	18056420	62.2226	5.99E-07
Chr25	14611429	556	14611413	56.5006	2.24E-06
Chr26	11594696	0	11594680	inf	0
Chr27	10985051	640	10985035	54.6506	3.43E-06
Chr28	10634025	28	10634009	68.0999	1.55E-07

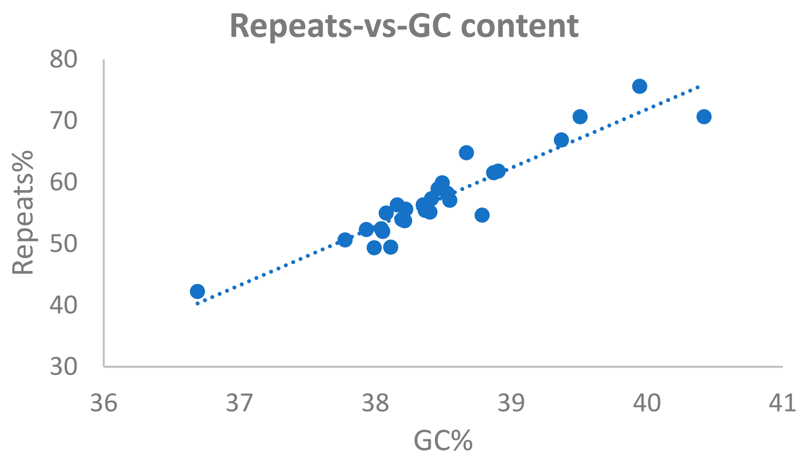

Figure S1 the content of repeats is positively correlated with GC content of each chromosome.
